# One-pot synthesis of sweetening syrup from lactose

**DOI:** 10.1038/s41598-020-59704-x

**Published:** 2020-02-17

**Authors:** Shouyun Cheng, Lloyd E. Metzger, Sergio I. Martínez-Monteagudo

**Affiliations:** South Dakota State University, Dairy and Food Science Department, Brookings, SD 57006 USA

**Keywords:** Heterogeneous catalysis, Heterogeneous catalysis, Chemical engineering, Chemical engineering

## Abstract

Lactose has become the main byproduct of many dairy products and ingredients. Current applications of lactose are insufficient to use the recovered lactose from manufacturing operations. Here we exemplified a new process for converting aqueous lactose into a sweeting syrup via one-pot synthesis. The synthesis consisted of two-steps: (1) enzymatic hydrolysis of lactose and (2) catalytic isomerization over MgO/SiO_2_. The hydrolysis of lactose over β-galactosidase converted 95.77 ± 0.67% of lactose into glucose and galactose. The catalytic isomerization was performed over MgO/SiO_2_ with different MgO loadings (10–40 wt.%). A battery of tests was conducted to characterize the different catalysts, including surface properties, basicity, and microstructure. The one-pot synthesis, enzymatic hydrolysis and catalytic isomerization over 20%-MgO/SiO_2_, converted 99.3% of lactose into a sweetening syrup made of glucose (30.48%), galactose (33.51%), fructose (16.92%), D-tagatose (10.54%), and lactulose (3.62%). The outcomes of this research present an opportunity for expanding the utilization of lactose.

## Introduction

Over the past few years, milk proteins have become a popular ingredient due to numerous health benefits associated with their consumption, including promoting satiety, appetite control, and exercise recovery^1^. As a result, milk protein-rich ingredients are commonly used to formulate beverage, snacks, desserts, and dietary supplements. Examples of milk protein ingredients are whey protein concentrate (WPC), whey protein isolate (WPI), whey protein hydrolysate (WPH), milk protein concentrates (MPC), milk protein isolates (MPI), and micellar casein concentrate (MCC). Specific details on the manufacture of milk protein ingredients can be found elsewhere^2^.

The manufacture of milk protein powders generates as the main by-product streams of lactose, whose concentration is 75–80% on a dry basis. Traditionally, lactose is concentrated and purified from streams derived from yogurt and cheese manufacture. Lactose is commercially available in a wide array of products for diverse uses, including infant foods, confectionery, and pharmaceutical application^3^. Despite such applications, the existing supply and demand for lactose are tight and traditional uses of lactose are insufficient to accommodate the recently increased production of lactose. Consequently, the prices of lactose have fallen to about $0.24–0.39 per pound, which is below its production cost. Expanding the utilization of lactose is not a trivial task due to its poor solubility, low sweetness, and malabsorption by specific population^4^. Purified lactose has been used as a feedstock for the production of lactitol, lactobionic acid, galactooligosaccharides, and others^5–7^.

A valuable ingredient that can be produced from lactose is D-tagatose, whose relative sweetness is 94% compared to that of sucrose, and its energy content is only 30% of the sucrose^8^. D-tagatose is an emerging sweetener and a potential replacer for sucrose and high fructose corn syrup, whose consumption has been related to the development of obesity. Fructose metabolism differs from the glucose, favoring the production of de novo lipogenesis^9^. Technological developments for the production of high fructose syrups can be found elsewhere^10^. D-tagatose has also been used as an additive in cosmetic, detergent, and pharmaceutical formulations^11^. Chemically, D-tagatose is an isomer of D-galactose, and it can co-exist as α-Dtagato-2,6-pyranose, β-D-tagato-2,6-pyranose, α-D-tagato-2,5-furanose, and β-D-tagato-2,6-furanose^12^. D-tagatose can be produced via extraction from some citrus, enzymatic conversion, or chemical synthesis. The extraction method is not economically viable because the concentration of D-tagatose is too low for potential commercialization^13^. Most of the D-tagatose commercialized in the world is produced via isomerization catalyzed L-arabinose isomerase using concentrated lactose or galactose. When lactose is used as a feedstock, an enzymatic treatment is first carried out to produce glucose and galactose. Oh^11^ reviewed reaction conditions and protein engineering on L-arabinose isomerase for the production of D-tagatose from galactose.

The chemical synthesis of D-tagatose is base-catalyzed isomerization, where the reaction is operated at high pH values using soluble alkalis (sodium hydroxide, potassium hydroxide, or calcium hydroxide)^14^, where D-galactose isomerizes via intermediate enolization according to the Lobry de Bruyn & Alberda van Ekenstein transformation^15^. The reaction is neutralized with H_2_SO_4_ once the desired level of conversion has been achieved. However, isomerization of D-galactose over base catalyst results in low selectivity towards the D-tagatose due to the formation of numerous by-products including the epimeric counterpart, furfural compounds, and organic acids^16^. Recent advances in the field of biorefinery have contributed to developing new classes of chemical catalysts that enable the interconversion of aldose to ketose^17^. A new class of catalysts of particular interest is those derived from Lewis acid, in which the active sites for the isomerization is embedded within the framework of an insoluble solid surface such as zeolites, hydrotalcites, resin, and others^18,19^. Earlier reports suggest that magnesium-based materials, including MgO, could be promising catalysts for the isomerization of sugars. Indeed, Murzin *et al*.^20^ studied the effect of MgO on the isomerization of glucose, galactose, and arabinose and reported relatively high conversions (36–76%). MgO offers the advantage of being applying in high amounts^21^. However, a major concern pointed out by Murzin *et al*.^20^ is the leaching of the MgO into the solution, where complete dissolution has been reported. Different inert supports such as multiwall carbon nanotubes, hydrotalcites, and aluminates have been used to increase the MgO stability. Among the support materials, SiO_2_ was found to be excellent neutral support for metal loaded catalysts^22^, and Sn/SiO_2_ catalysts showed high conversion of fructose during the glucose isomerization^23^. To the best of our knowledge, no study has been reported on the use of MgO/SiO_2_ catalysts for the isomerization of hydrolyzed lactose solution. Thus, the objective of this study was to develop a one-pot synthesis for the production of sweetening syrup from lactose aqueous solution.

## Results and Discussion

### Lactose hydrolysis

**Figure 1:**
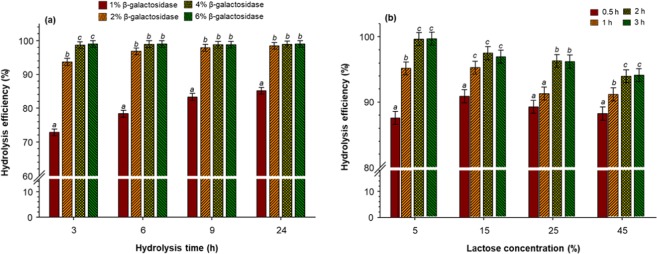
Hydrolysis of lactose: (**a**) 1, 2, 4, and 6% of β-galactosidase after 3, 6, 9, and 24 h using 5% of lactose concentration; (**b**) 5, 15, 25, and 45% of lactose after 0.5, 1, 2, and 3 h of hydrolysis using 4% of β-galactosidase. Reaction temperature = 25 °C and agitation = 1200 rpm.

The first set of screening experiments for the enzymatic hydrolysis of lactose is shown in Fig. 1a. When the hydrolysis was performed using 1% of β-galactosidase, the hydrolysis efficiency gradually increased with the hydrolysis time reaching values of 85% after 24 h. The hydrolysis efficiency achieved values of 95% within the first 6 h of hydrolysis with 2% of the enzyme, and afterward, the hydrolysis increased slightly to reach values of 98%. At 4 and 6% of enzyme concentration, the hydrolysis efficiency was more than 98% regardless of the time. Concentrations of enzyme higher than 4% and hydrolysis time longer than 3 h does not substantially increased the hydrolysis efficiency. Thus, the second set of screening experiments consisted in evaluating the effect of lactose concentration at a constant concentration of enzyme (4%) on the hydrolysis efficiency (Fig. 1b). The hydrolysis efficiency increased with time and decreased with the concentration of lactose. At concentration levels of 25 and 45%, it was required 2 and 3 h of hydrolysis to achieve at least 95% of efficiency. Contrary, only 1 h was needed to achieve 95% at concentration levels of 5 and 15%. The concentration of lactose in the dairy by-product streams can vary from 5 to 18%, depending on the concentration steps. Thus, subsequent experiments were conducted using a lactose solution of 15% hydrolysed at 1 h using 4% of β-galactosidase.

### Surface properties of catalysts

The nitrogen adsorption-desorption isotherms of the SiO_2_ and the prepared MgO/SiO_2_ were determined to evaluate the surface properties of the catalysts. Representative isotherms are provided in the Supplementary Information (Fig. S1). For the SiO_2_, the adsorbed volume increased with the relative pressure (P/Po) in a sigmoidal fashion, resembling a Type-IV isotherm with an H1 broad hysteresis loop according to the IUPAC classification^24^. Interestingly, the addition of MgO into the SiO_2_ frame neither alters the shape nor the hysteresis loop of the isotherms. All isotherms display a sharp increase in the adsorbed volume at relative pressure of 0.5–0.6, and a hysteresis loop from 0.5 to 0.9 of relative pressure. These are characteristics of mesoporous materials containing 1-D cylindrical channels where nitrogen condensates. On the other hand, the sharp increase in the adsorbed volume is an indication of capillary condensation of nitrogen across the SiO_2_ surface.

The surface properties of the pure SiO_2_ and MgO/SiO_2_ are listed in Table 1. The specific surface area (S_BET_) decreased gradually from 336.05 to 100.05 m^2^ g^−1^, with the loading of MgO from 0 to 40%. Similarly, the total pore volume (V_pore_) decreased from 0.69 to 0.36 cm^3^ g^−1^ with increased loading of MgO. Such changes are due to the deposition of MgO molecules within the surface of the SiO_2_ support^25,26^. Concurrently, changes in S_BET_ and V_pore_ were accompanied by an increase in the d_pore_ from 8.17 to 14.39 nm. The increase in the d_pore_ was caused by voids between particles, which resulted from MgO aggregates deposited on the external surface of SiO_2_. These observations indicate the incorporation of metal atoms into the SiO_2_ framework^27^.

**Table 1:** Surface properties of different MgO/SiO_2_ catalysts.

Catalyst	S_BET_ (m^2^ g^−1^)	V_pore_ (cm^3^ g^−1^)	d_pore_ (nm)
SiO_2_	336.05	0.69	8.17
10-MgO/SiO_2_	290.27	0.68	9.63
20-MgO/SiO_2_	205.10	0.66	13.05
30-MgO/SiO_2_	125.21	0.43	13.94
40-MgO/SiO_2_	100.05	0.36	14.39

S_BET_ – BET surface area; V_pore_ – total pore volume; d_pore_ – average pore size.

The prepared catalysts were analysed by XRD, and representative patterns are provided in Supplementary Information (Fig. S2). For all catalysts, a single broad diffraction peak located at about 22.3° was detected, which corresponds to the amorphous SiO_2_ (JCPDS 29–0085). Similarly, Jia *et al*.^28^ reported a broad diffraction peak centered at around 22.0° for calcined SiO_2_. Interestingly, no diffraction peaks were detected for MgO regardless of the load, a phenomenon that has been explained due to highly dispersed MgO species on the surface of the catalyst^29^. The presence of a single broad peak indicates that the crystalline structure of SiO_2_ was maintained after MgO impregnation.

**Figure 2:**
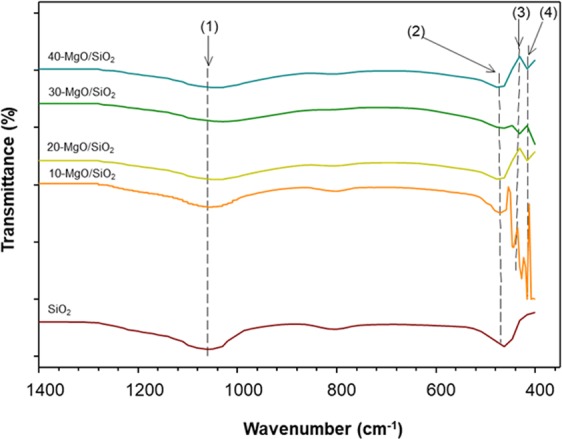
FTIR spectra of MgO/SiO_2_ with different MgO loadings. Arrows (1) and (2): Si-O-Si; arrows (3) and (4) Mg-O.

The surface characteristics of the prepared catalysts (SiO_2_ and MgO/SiO_2_) were also investigated by Fourier Transform Infrared spectroscopy (Fig. 2). All the catalysts exhibited two bands, represented by (1) and (2) in Fig. 2. The first band at 1030 cm^−1^ corresponds to the stretching vibration of Si-O-Si, and the second band at 458 cm^−1^ corresponds to the stretching and deformation modes of the Si-O-Si framework. Similar FTIR spectra for SiO_2_ have been reported elsewhere^30,31^. On the other hand, the presence of Mg-O was revealed in MgO/SiO_2_ by two additional bands, (3) and (4) in Fig. 2, at 423 and 413 cm^−1^. Similar bands have been reported elsewhere^31,32^. The FTIR spectra supported our claim of the successful introduction of MgO to SiO_2_ support.

**Figure 3:**
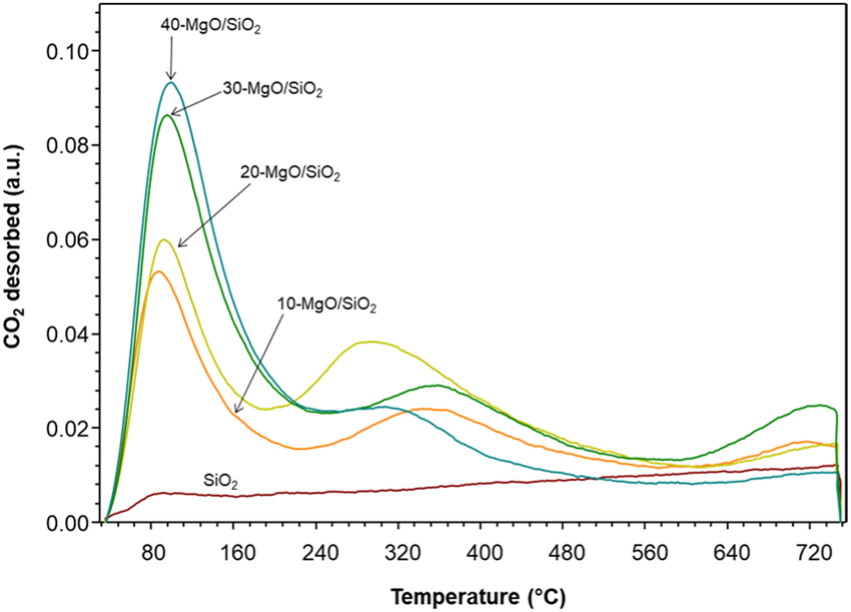
CO_2_-TPD curves of MgO/SiO_2_ with different MgO loadings.

The catalysts were also evaluated for their basicity using CO_2_-TPD experiments (Fig. 3). The MgO/SiO_2_ spectra showed three distinctive desorption peaks at around 30–160, 160–400, and >600 °C indicating desorption of CO_2_ from weak, medium, and strong basic sites, respectively^33^. In comparison, the parent SiO_2_ displayed only a small desorption peak corresponding to region of weak basic sites. The incorporation of MgO enhanced the basicity of MgO/SiO_2_ catalyst compared to SiO_2_. The desorption peaks that span from 30 to 160 °C is mainly due to interaction of CO_2_ and hydroxyl groups on the surface of MgO/SiO_2_. On the other hand, the desorption peaks spanning from 160 to 400 °C is attributed to the association of CO_2_ with basic Mg^2+^ and O^2−^ pairs. As the concentration of MgO increases in the MgO/SiO_2_, the desorption peaks of CO_2_ become more intense and partially shift to a higher total area in the region of weak basic sites, indicating that the amount and strength of the basic sites increased in with the MgO loading. Similarly, She *et al*.^34^ reported that the incorporation of Mg species enhances the basicity of the catalyst. The CO_2_ adsorption curves for 10-, 20-, and 30-MgO/SiO_2_ displayed a third peak at temperatures higher than 600 °C, which corresponds to the presence of strong and isolated basic sites O^2−^ ^33^. In summary, CO_2_- TPD curves (Fig. 3) revealed that MgO/SiO_2_ is mostly characterized by weak basic hydroxyl groups, followed by medium basic sites such as Mg^2+^ and O^2−^ pairs.

**Figure 4:**
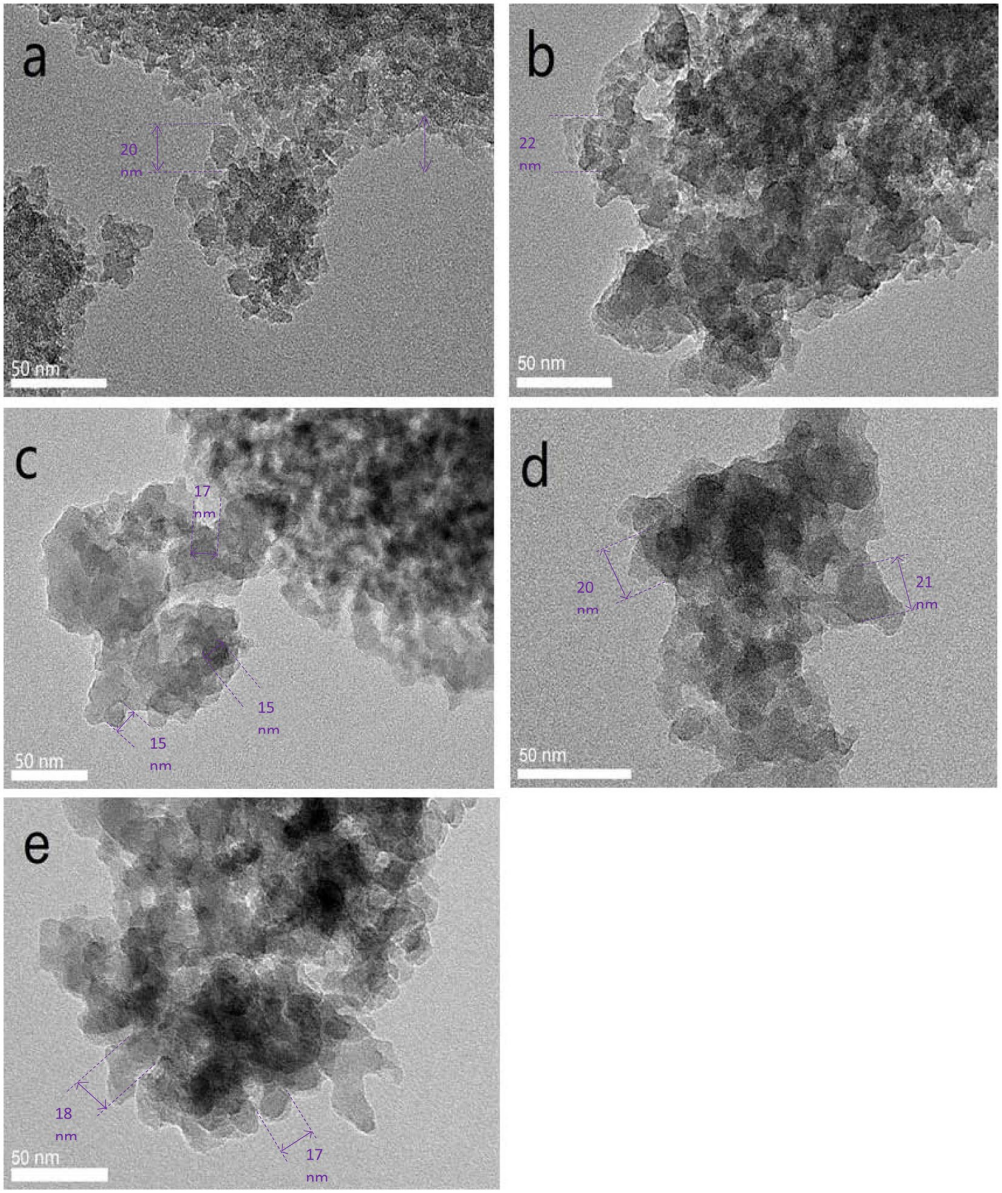
TEM micrographs of the prepared catalyst: (**a**) SiO_2_; (**b**) 10-MgO/SiO_2_; (**c**) 20-MgO/SiO_2_; (**d**) 30-MgO/SiO_2_; and (**e**) 40-MgO/SiO_2_.

Representative TEM images of SiO_2_ and MgO/SiO_2_ containing different loadings of MgO are provided in Fig. 4. For SiO_2_, an amorphous-like structure of mesoporous can be observed, having an approximate size of 20 nm (Fig. 4a). The presence of MgO was confirmed in the TEM images by the existence of dark spots within the amorphous structure of SiO_2_, which has an approximate size of 15–22 nm (Fig. 4b–e). Such dark spots were attributed to the presence of MgO particles. Similar dark spots arising from MgO have been reported in TEM images^35^. It seems that MgO species are embedded within the amorphous structure of SiO_2_, and the morphology of MgO cannot be clearly visualized.

### Catalytic isomerization

**Figure 5:**
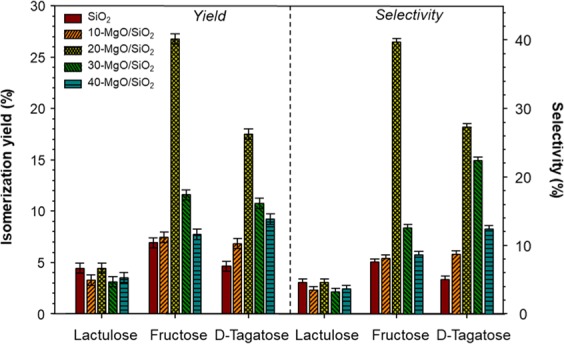
Reaction products yield derived from the enzymatic hydrolysis of lactose, followed by the catalytic isomerization over different MgO/SiO_2_. Hydrolysis conditions: 10 g of 15wt.% lactose solution, 4% β-galactosidase, 25 °C, 1 h, 1200 rpm. Isomerization conditions: 10 g of 15wt.% lactose hydrolysis solution, 0.15 g of catalysts, 110 °C, 2 h, 1200 rpm.

Glucose, galactose, and the remaining lactose produced during the enzymatic hydrolysis were used as a starting material for the second step, catalytic isomerization over MgO/SiO_2_. Figure 5 shows the isomerization yield and selectivity for fructose, D-Tagatose, and lactulose. The remaining lactose after hydrolysis (<5%) underwent isomerization to produce lactulose with rather low yield values (3–4%) regardless of the catalysts used. Similarly, the selectivity of lactulose was relatively low, ranging from 3–5%. These low values are not surprising since the initial concentration of lactose was too low compared with the initial concentration of glucose and galactose (47% each). For the formation of fructose, the isomerization yield increased with the MgO loading, reaching a maximum value of 26.1 ± 0.5% at 20-MgO/SiO_2_. Further increase of the MgO loading results in yields of 11.6 ± 0.61 and 7.7 ± 0.7% at 30- and 40-MgO/SiO_2_. Similar results have been reported elsewhere. Delidovich and Palkovits^17^ reviewed the catalytic isomerization of glucose to produce fructose using different heterogeneous catalysts and reported yield values from 19–37%. Similar tendencies were observed for the selectivity toward fructose, where 20-MgO/SiO_2_ produced the highest selectivity, 40%. The isomerization of galactose to produce D-Tagatose is also shown in Fig. 5.

The highest isomerization yield (17.5 ± 0.7%) was obtained with 20-MgO/SiO_2_ followed by 30-MgO/SiO_2_ (10.7 ± 0.8%), while the other catalyst (SiO_2_, 10- and 40-MgO/SiO_2_) produced yields in the range of (4.2–9.2%). The incorporation of MgO improved the overall basicity, which favors the isomerization of aldoses, where an extraction of a proton from the carbonyl group occurred. Indeed, Yu, *et al*.^19^ reported that the isomerization of glucose is favored in the presence of weak sites as opposed to medium and strong basic sites. The CO_2_-TPD curves provide a qualitative measure of the catalytic activity of MgO/SiO_2_. The CO_2_-TPD profile of SiO_2_ showed rather a small basicity peak, which explains the low isomerization yield. The CO_2_-TPD of 20-MgO/SiO_2_ showed two peaks of similar size, namely weak and medium basic sites. On the other hand, TEM images (Fig. 4c) for 20-MgO/SiO_2_ showed dark spots of small size, an indication of well-dispersed MgO particles across the surface of SiO_2_, which may improve the catalytic performance. The improved catalytic performance of 20-MgO/SiO_2_ may be due to the weak and medium basicity and highly dispersed MgO within the SiO_2_ support. The 20-MgO/SiO_2_ catalyst was the most effective catalyst for the production of fructose (26.8 ± 0.5%) and D-Tagatose (17.5 ± 0.5%). On the other hand, CO_2_-TPD of 40-MgO/SiO_2_ showed a large peak in the range of weak sites, which one would expect isomerization yields higher than ~7%. Leaching of MgO into the soluble phase cannot be ignored, as previous researchers have pointed out^20^. A yellowish color was clearly visible in the samples catalyzed with 40-MgO/SiO_2_, and the pH of the slurry increased from 6.68 to 7.81. These two observations may indicate some degree of MgO leaching that could explain the rather low catalytic activity of 40-MgO/SiO_2_. However, a systematic evaluation of leaching will confirm such claims.

### One-pot synthesis

**Figure 6:**
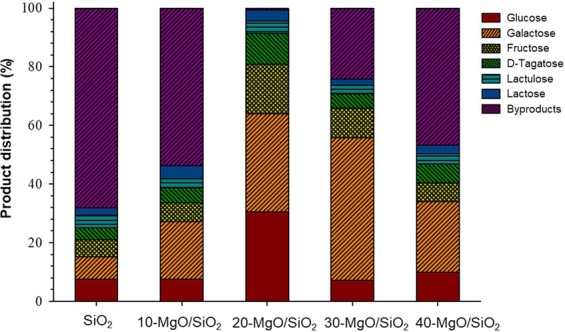
The overall products yield derived from the enzymatic hydrolysis of lactose followed by the catalytic isomerization over different MgO/SiO_2_. Hydrolysis conditions: 10 g of 15wt.% lactose solution, 4% β-galactosidase, 25 °C, 1 h, 1200 rpm. Isomerization conditions: 10 g of 15wt.% lactose hydrolysis solution, 0.15 g of catalysts, 110 °C, 2 h, 1200 rpm.

Figure 6 shows the concentration of the different carbohydrates produced after the isomerization over MgO/SiO_2_. The concentration of such products was influenced by the catalyst used. A syrup made of 6 carbohydrates (glucose, galactose, fructose, D-tagatose, lactulose, and remaining lactose) was by the application of one-pot synthesis. In the first step, 95.77 ± 0.67% of the lactose was converted into glucose and galactose, while the catalytic isomerization over 20-MgO/SiO_2_ converted 99.3% of lactose into glucose (30.4%), galactose (33.5%), fructose (16.9%), D-tagatose (10.5%), and lactulose (3.6%). Remarkably, only 0.6% of byproducts were formed using 20-MgO/SiO_2_, while 24–68% were formed with the other catalysts (SiO_2_, 10-, 30-, and 40- MgO/SiO_2_). This is a promising observation indicating that 20-MgO/SiO_2_ can be a suitable catalyst to convert lactose streams into sweetening syrup. The concentration of the final products may be further optimized according to their kinetics. The hexoses formed through the one-pot synthesis of lactose are of industrial relevance, and their individual production methods have been recently reviewed elsewhere^36^.

### Reaction mechanism of lactose hydrolysis and isomerization

**Figure 7:**
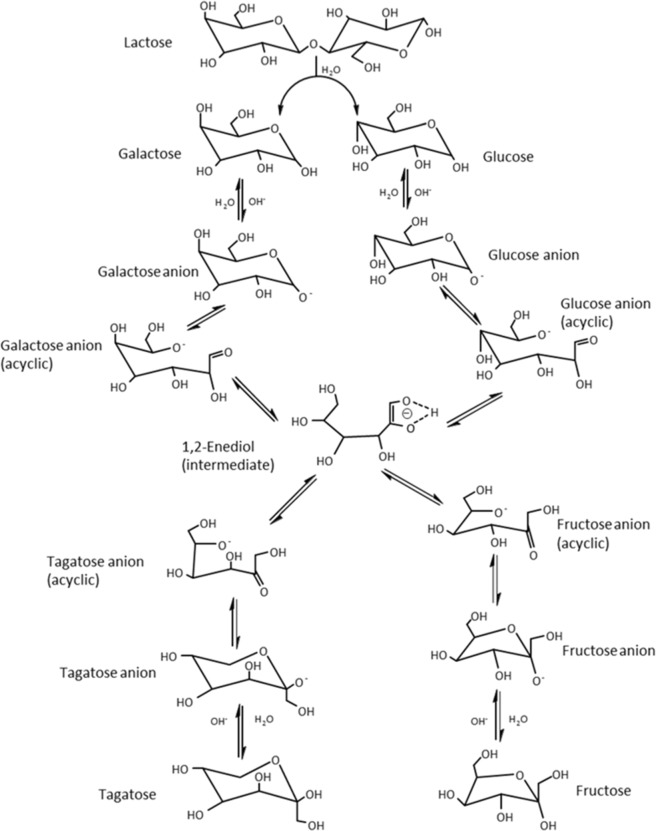
Proposed reaction mechanism for the production of D-Tagatose and fructose from lactose using enzymatic hydrolysis followed by catalytic isomerization over MgO/SiO_2_.

A proposed reaction mechanism for the production of a sweeting syrup made of glucose, galactose, fructose, and D-tagatose via two-step process consisting of enzymatic hydrolysis and catalytic isomerization is illustrated in Fig. 7. Firstly, the lactose was hydrolyzed to produce galactose and glucose in the presence of β-galactosidase (Ha-Lactase 5200). Then, the galactose was converted to galactose anion after the abstraction of an anomeric proton in the aqueous solution. Then, the galactose anion is transformed into galactose anion (acyclic) through ring-opening and further isomerized to an intermediate compound of 1,2-endiol. In the presence of basic MgO/SiO_2_, the tagatose anion is formed due to the shift of a proton from the OH group attached to the second carbon atom to the first carbon atom of the endiol and a ring closure in the endiol structure^14^.

The tagatose anion is converted to tagatose through the acceptance of a proton from the aqueous solution. On the other hand, the glucose was converted to glucose anion after the absorption of an anomeric proton from the aqueous solution. Then, the glucose anion was isomerized to glucose anion (acyclic) through ring-opening and further changed into the intermediate 1,2-endiol in the presence of basic MgO/SiO_2_ catalyst. After that, the 1,2-endiol is isomerized to fructose anion through proton shifting and ring-closing^37^. Finally, fructose is produced from fructose anion through the absorption of a proton from the aqueous solution.

In closing, the one-pot approach was used to convert aqueous lactose into a mixture of carbohydrates glucose, galactose, fructose, D-tagatose, and lactulose. The MgO/SiO_2_ catalysts with different MgO loading ratios (10, 20, 30, and 40 wt.%) were characterized and used in the isomerization at 110 °C for 2 h. Overall, the one-pot synthesis converted 99.3% of lactose into a sweetening syrup made of glucose (30.48%), galactose (33.51%), fructose (16.92%), D-Tagatose (10.54%), lactulose (3.62%), and byproducts (0.69%). The reaction mechanism of lactose hydrolysis and isomerization to produce tagatose and fructose in the presence of MgO/SiO_2_ catalysts was proposed.

## Material and Methods

### Materials

D-(+)-glucose (≥99%), D-(+)-galactose (≥99%), and silica gel (technical grade, SiO_2_) were purchased from Sigma-Aldrich (St. Louis, MO). α-D-Lactose monohydrate (≥98%) and magnesium nitrate hexahydrate (98%, Mg(NO_3_)_2_·6H_2_O) were purchased from Acros Organics, while D-(−)-fructose (≥99%), D-(−)-Tagatose (≥99%), and lactulose (≥99%) were obtained from Fischer Scientific (Hampton, NH). The enzyme β-galactosidase (Ha-Lactase 5200) was donated by Chr. Hansen (Milwaukee, WI).

### Catalyst preparation

A set of MgO/SiO_2_ with different MgO loadings were prepared using the method of wet impregnation. The SiO_2_ was added to a predetermined amount of aqueous solution of Mg(NO_3_)_2_·6H_2_O corresponding to 10, 20, 30, and 40 wt./wt.% MgO loading ratios. Then, the mixture was stirred for 4 h at room temperature allowing the impregnation to occur. Afterward, the solution was dried in the presence of air at 120 °C for 12 h. Finally, the MgO/SiO_2_ catalyst was calcinated at 500 °C for 5 h. The obtained catalysts were named as x-MgO/SiO_2_, being x the percentage of MgO incorporated.

#### Catalysts characterization

The specific surface area (S_BET_), total pore volume (V_pore_), and pore size (d_pore_) of the prepared catalyst were calculated from the nitrogen adsorption data according to the Brunauer-Emmett-Teller (BET) methodology. The nitrogen adsorption measurements were acquired using a Micromeritics instrument (automatic Micromeritics ASAP 2020) operated at 77.2 K according to the methodology reported elsewhere^22^.

XRD measurements were conducted using an X-ray diffractometer (Rigaku Corporation, MiniFlex) operated at 35 kV and 15 mA, following the method reported by Cheng, *et al*.^22^. Samples were scanned from 10° to 80° using filtered Cu-Kα radiation. The scan speed and step size were set as 2° min^−1^ and 0.02° (2 θ), respectively.

Basicity of MgO/SiO_2_ (10–40%, wt./wt.) was measured through CO_2_-temperature programmed desorption (CO_2_-TPD) using a Chemisorption Analyzer (Micrometrics Autochem II) with a thermal conductivity detector. The method reported was used by She *et al*.^34^. Briefly, 0.3 g of catalyst was dried at 120 °C for 12 h, followed by rapid cooling to 25 °C. Then, the dried catalyst was purged with CO_2_ for 4 h, and purged with helium at a constant flow of 60 mL min^−1^ at 25 °C for 2 h. Samples were heated from 25 to 750 °C at a constant rate of 10 °C min^−1^ and held at 750 °C for 1 h.

Fourier transform infrared spectroscopy (FTIR) spectrum of MgO/SiO_2_ catalysts were recorded at 0.01 cm^−1^ resolution by a Nicolet 380 ATR-FTIR spectrophotometer (Thermo Electron Corporation). Each spectrum was an average of 100 scans^38^.

TEM images of catalyst samples were recorded by a JEOL JEM-2100 LaB6 transmission electron microscope that was operated at 200 kV. Catalysts were dispersed into isopropyl alcohol before the test. The solution was then shaken in ultrasound for several minutes. Then, the suspension droplets were loaded and dried on a carbon-coated copper grid.

### Enzymatic hydrolysis

A set of screening experiments was conducted to down select the hydrolysis conditions. Firstly, the effect of the enzyme concentration (1, 2, 4, and 6%) and hydrolysis time (3, 6, 9, and 24 h) on the conversion of lactose was evaluated at a constant concentration of lactose. Secondly, the effect of lactose concentration (5–45%) and hydrolysis time (0.5, 1, 2, and 3 h) was evaluated at a constant concentration of enzyme. All the experiments dealing with the enzymatic hydrolysis were conducted at room temperature (25 °C) under continuous mixing using a magnetic stirrer at 1200 rpm. After a predetermined hydrolysis time, the samples were removed and cooled by ice water to prevent further hydrolysis. The hydrolysis efficiency was evaluated using Eq. (1).

1 $$Hydrolysis\,efficiency\,( \% )=\frac{initial\,mole\,of\,lactose-mole\,of\,unreated\,lactose}{intial\,mole\,of\,lactose}\times 100$$

### Isomerization over MgO/SiO_2_

Upon completion of the enzymatic hydrolysis, 0.15 g of catalyst was added to the hydrolyzed lactose solution. The tested catalysts were SiO_2_, 10-, 20-, 30-, and 40-MgO/SiO_2_. The aqueous hydrolyzed solution containing the catalyst was evacuated and pressurized with N_2_ at 14 psi. Then, the catalytic isomerization was evaluated at 110 °C for 2 h. At the end of the reaction, the vials were cooled down to room temperature using ice water. Samples were filtered to remove the used catalysts, and the remaining liquid containing the reaction products was stored at −20 °C until further analysis.

#### Quantification of reaction products

The products derived from the enzymatic hydrolysis were analyzed by HPLC following the methodology described elsewhere^39^. The analysis of the reaction products from the catalytic isomerization (D-Tagatose, fructose, lactulose, glucose, galactose, and residual lactose) was performed on a Shimadzu LC system (LC-20AD, Shimadzu Corp) combined with a Qtrap 5500 triple quadrupole mass spectrometer (AB Sciex). Details of the methodology can be found elsewhere^40^.

### Data analysis

The overall product yield was calculated with respect to the initial concentration of lactose, according to Eq. (2). The product yield for fructose and D-tagatose was calculated as an isomerization yield ($${Y}_{is}$$) according to Eq. (3). The selectivity toward fructose ($${S}_{Fru})$$ and D-tagatose ($${S}_{Tag})$$ was calculated with Eq. (4). Experimental runs were conducted in triplicates, and all figures were made using Sigmaplot software V11 for Windows (SPSS Inc., Chicago, IL, USA).

2 $${Y}_{ov}=\frac{mole\,of\,product}{Initial\,mole\,of\,lactose\,}\times 100$$

3 $${Y}_{iso}=\frac{mole\,of\,desired\,product\,}{mole\,of\,the\,corresponding\,sugar}\times 100$$

4 $${S}_{i}=\frac{Yield}{Conversion}\times 100$$

## Supplementary information

Supplementary information.

## Data Availability

The data generated during the current study are available from the corresponding author upon reasonable request.
