# Supplementary material for: One-pot synthesis of sweetening syrup from lactose
Source: Sci Rep. 2020 Feb 17;10:2730. doi: 10.1038/s41598-020-59704-x (PMC7026174; doi:10.1038/s41598-020-59704-x)
Supplement: Supplementary file 1 — Supplementary information. [file 41598_2020_59704_MOESM1_ESM.docx]

**Supporting information SREP-19-28125-TA**

**One-pot synthesis of sweetening syrup from lactose**

Shouyun Cheng ^1^, Lloyd E. Metzger ^1^, and Sergio I. Martínez-Monteagudo ^1^*

^1^South Dakota State University, Dairy and Food Science Department, Brookings, SD 57006, USA

Supplementary material content:

- **Figure S1.** Nitrogen adsorption-desorption isotherms of MgO/SiO_2_ with different MgO loadings.
- **Figure S2**. XRD patterns of MgO/SiO2 with different MgO loadings.

**Figure S1.** Nitrogen adsorption-desorption isotherms of MgO/SiO_2_ with different MgO loadings.

**Figure S2**. XRD patterns of MgO/SiO2 with different MgO loadings.
